# The central role of chromatin maintenance in aging

**DOI:** 10.18632/aging.100106

**Published:** 2009-12-09

**Authors:** Gianluca Pegoraro, Tom Misteli

**Affiliations:** National Cancer Institute, NIH, Bethesda, MD 20892, USA

**Keywords:** chromatin, DNA repair, progeria, histones, epigenetics

## Abstract

Epigenetic regulation of chromatin and the DNA damage response are now well appreciated key players in human aging. What contributions chromatin and DAN repair make to aging, whether they are causal, and how these relate to other aging pathways, however, is unclear. Novel insights into the aging-related molecular mechanisms that link chromatin and DNA damage repair have recently been gained by studying models of both premature and physiological aging. Here we discuss these findings and we propose a broad framework for the role of chromatin in aging to reconcile apparently contradicting evidence obtained in various experimental systems.

## Introduction

Cells are continuously exposed to a wide variety of physical and chemical stresses such as oxidation, radiation and heavy metals, which cause damage to cellular proteins, lipids and DNA. Organisms have evolved multiple protective mechanisms to counteract these endogenous and exogenous damages. Nevertheless, the effectiveness of these protective pathways seems to decline with age. As such, aging can be defined as the decrease in the probability of successful repair of cellular damage.

One of the major sources of cellular insults is damage to DNA. To counteract detrimental DNA damage, cells are endowed with a complex network of DNA damage response (DDR) proteins which are capable of detecting DNA damage, and then triggering and amplifying a signaling cascade, which ultimately leads to either cell-cycle arrest and DNA repair, or to apoptotic cell death to eliminate permanently damaged cells [1]. The importance of the DDR in maintaining genomic integrity and limiting the effects of aging is highlighted by premature aging phenotype of mice that lack key DNA repair factors [2]. More tellingly, almost all genetic conditions that lead to premature aging in humans have been mapped to genes belonging to the DDR [3]. Mutations in the Werner DNA helicase, which is required for DNA replication and at telomeres, lead to Werner syndrome and the components of the nucleotide excision repair (NER) XPC, ERCC6, ERCC8 and the ERCC1/XPF complex involved in inter-strand DNA crosslink repair are mutated in Cockayne Syndrome and in Trichothiodystrophy (TTD), two prominent premature aging disorders [4]. These findings suggest a prominent, and causal, role for DNA damage responses in aging.

The DDR, like all major nuclear processes such as DNA replication and transcription, operates in the context of the chromatin fiber [5,6]. Chromatin is made up of nucleosomes, repetitive units of 146bp of DNA tightly wrapped around an octameric core of histone proteins (H2A, H2B, H3 and H4). Nucleosomes are further packaged into higher order structures by the action of architectural chromatin proteins such as histone H1 and heterochromatin protein HP1.  Based on cytological criteria, chromatin is classified into euchromatin, which is loosely packed and generally transcriptionally active, and into heterochromatin, which is more compacted and generally represents a transcriptionally repressive environment. Nucleosomal histones are modified by complex patterns of post-translational modifications (PTM) such as acetylation, methylation and ubiquitination which appear to dictate the dynamic recruitment of non-histone proteins to chromatin and regulate its function [7]. Furthermore, chromatin structure and function is also determined by the methylation status of DNA itself and by a large number of ATP-dependent remodeling factors. Both the level of chromatin compaction, and hence the accessibility of DNA, and the recruitment of chromatin-associated factors determine the outcome of transcription, DNA replication and DNA damage repair. All these modifications to chromatin structure, and thus its informational content, are inherited through several cycles of cell division and as such represent an epigenetic memory [8].

### Chromatin defects in aging

Chromatin defects are associated with aging. The first hints pointing to a possible link between chromatin maintenance and aging came from studies in the yeast S. *cerevesiae*, where the NADH-dependent Sir2 histone deacetylase Sir2 was found to be important for establishing heterochromatin at telomeres, at ribosomal DNA (rDNA), and at HMR and HMR loci, which encode factors needed for yeast mating type switching [9-14]. Upon prolonged growth, equated to aging in yeast, repetitive rDNA tends to hyper-recombine and form extrachromosomal rDNA circles (ERC), indicative of increased chromatin fragility [15]. Formation of heterochromatin at rDNA sites by overexpression of Sir2 reduces this hyper-recombination and prolongs lifespan, suggesting a contribution of chromatin structure to aging [16]. Further experiments in worms and flies demonstrated a similar role in lifespan extension for Sirt1, the closest orthologue of yeast Sir2 in these organisms [17,18]. Nevertheless, the role of Sirt1 in increased longevity in higher eukaryotes might not just involve heterochromatin maintenance, since in this case the molecular mechanism does not seem to involve ERC stabilization [19]. Furthermore, the analysis is complicated by the fact that in mammals SIRT1 deacetylates a wide variety of non-histone, aging-related transcription factors such as p53, HSF1 and members of the FOXO transcription factors family [20-22]. Identification of the mechanisms of action of SIRT1 in higher organisms will be key to clarifying its role in the aging process.

There are several other clear indications for a role  of chromatin and its maintenance in aging. A hallmark of cellular aging is the appearance of characteristic changes in the epigenetic make-up of the genome.  Epigenetic changes associated with aging in mammalian cells include loss of DNA methylation at repetitive DNA sequences [23-25], which are generally heterochromatinized, and an increase in DNA methylation at CpG islands in the promoters of specific genes [26,27]. Cells from aged individuals and patients with the premature aging disorder Hutchinson-Gilford Progeria Syndrome (HGPS) are also characterized by loss of heterochromatin, by loss of key architectural chromatin proteins such as HP1 and the histone mehtyltransferase EZH2, and, importantly, by alterations in the levels of heterochromatin-associated histone PTM including H3K9me3 and H3K27me3 [28-31]. Interestingly, both prematurely and normally aged cells exhibit dramatically increased levels of unrepaired DNA damage [30,32].

In addition to epigenetic and structural chromatin defects, there are indications that aging in mammals is accompanied by stochastic deregulation of gene expression.  Transcriptional noise at the single cell level increases with age in the mouse heart, most likely as a consequence of oxidative DNA damage [33]. Furthermore, in mammalian cells oxidative DNA damage also seems to relocalize SIRT1 from otherwise transcriptionally repressed genes to sites of DNA damage [34]. This has led to the speculation that, through unknown mechanisms, aging disrupts the epigenetic organization of heterochromatin both at a global and at a gene-specific level, thus leading to elevation of stochastic transcriptional noise and to the disruption of transcriptional programs necessary for proper cell homeostasis [35]. In contrast to this model of stochastically occurring defects in gene expression programs, the aging process seems to also induce a specific transcriptional response, which dampens the somatotrophic IGF-1 axis and helps protecting cells from DNA damage and stress [36].

The study of chromatin in aging also points to a key influence of aberrant chromatin structure on aging-related defects in DNA repair. Impairment of SIRT1 leads to defective DNA damage repair in mammalian cells [34] and a knock-out mouse model for SIRT6 shows signs of premature aging and has defects in the base excision repair pathway [37]. The exact molecular basis for these phenotypes is not clear yet. One possibility is that SIRT6 affects genomic stability by regulating the levels of H3K56Ac [38,39], a PTM important for chromatin assembly and DNA damage tolerance in yeast [40,41].

### A molecular mechanism for aging-associated chromatin defects

The molecular mechanisms leading to chromatin defects in aging are largely unknown. Recent analysis of chromatin defects in the premature aging disease HGPS have given some of the first insights into how chromatin ages [42]. HGPS is an extremely rare genetic disease caused by a *de novo* point mutation in the lamin A (LMNA) gene, a major structural component of the nuclear envelope [43].The pathogenic mutation leads to the production of an internally truncated form of lamin A, referred to as progerin. This protein acts in a dominant-negative gain of function fashion causing the diverse and pronounced chromatin defects. Analysis of the molecular mechanisms involved in bringing about chromatin defects in HGPS and old cells uncovered the NURD complex as a key player in aging [42]. NURD is a ubiquitous chromatin remodeling complex which contains the histone deacetylases HDAC1 and HDAC2 and the ATPases CHD3 and CHD4 as catalytic subunits. NURD has been implicated in transcriptional repression at specific promoters and more recently has also been shown to associate with pericentromeric heterochromatin [44,45]. The protein levels and the activity of several NURD components including HDAC1 and the histone chaperones RBBP4/, are reduced in HGPS cells and normally aged cells. A direct role for NURD loss in aging-associated chromatin defects is indicated by the finding that knock-down of NURD members in normal cells recapitulates aging-related chromatin defects including heterochromatin loss and increased DNA damage [42]. NURD is known to be involved in a variety of chromatin functions and its loss may explain the broad spectrum of chromatin defects seen in aged cells [42].

### Chromatin structure as a trigger of aging

**Figure 1.**
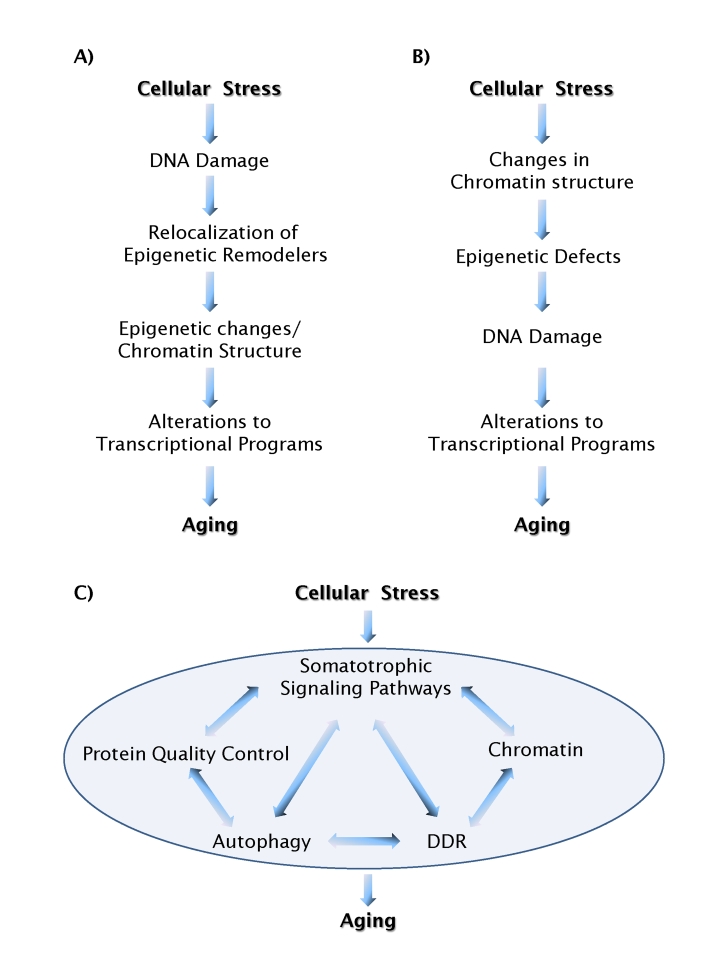
Models of aging pathways. (**A**) A scenario in which DNA damage acts as a causal trigger for aging. (**B**) A scenario in which chromatin structure acts as a causal trigger for aging. Feedback loops, which are likely to exist between most individual events, are not shown for simplicity.  (**C**) Chromatin structure and DNA damage pathways act in an integrated fashion with a multitude of other cellular process to form a network of aging processes.

There is little doubt that chromatin defects and DNA damage play a part in the aging process. The unresolved question is: how? One recently proposed scenario suggests that DNA damage and the cellular response to it leads to chromatin defects via relocation of epigenetic machinery from its normal distribution in the genome and to structural chromatin changes, eventually resulting in gene misregulation [34] (Figure 1A). An alternative possibility is that the aging process is triggered by loss of chromatin structure, leading to altered epigenetic modifications, and increased susceptibility to DNA damage. In this model DNA damage is a downstream event (Figure 1B). The key question to distinguish between these two models is: what comes first, DNA damage or chromatin defects? A partial answer comes from recent observations in the premature aging disorder HGPS. Upon induction of the dominant negative disease-causing protein in normal skin fibroblasts, chromatin defects occurred prior to DNA damage [42][41]. Further support for a trigger role of chromatin structure in DNA damage and aging, is the observation that suppression of the activity of chromatin modifiers generates high levels of endogenous DNA damage, as seen in the case of several subunits of the NURD complex [42], the SET8 H4K20 histone methylase [46,47], and for the Su(var)3-9 H3K9 histone methylase in *Drosophila *[48]. In these cases chromatin structural defects clearly precede DNA damage, placing epigenetic and chroma-tin structure changes upstream of DNA damage events.

How may aberrant chromatin structure lead to DNA damage and aging? Although only poorly investigated and understood, it is becoming clear that chromatin structure affects the susceptibility of DNA to damage and progression of the DDR [5]. DNA repair occurs with slower kinetics in highly condensed heterochromatin, presumably due to the inability of repair factors to rapidly access the site of damage [49]. Furthermore, heterochromatinized regions of the genome, like nucleoli, centromeres and telomeres tend to be rich in repetitive sequences that are particularly prone to recombination. As such it is possible that the compacted nature of heterochromatin suppresses hyper-recombination of repetitive sequences, the formation of aberrant DNA structures and genomic instability [50]. Another, not-mutually exclusive, possibility is that altered chromatin structure increases the steady-state level of DNA damage due to replication defects such as impaired passage of the replication machinery or to replication fork stalling. It is indeed possible that intact heterochromatin conformation is necessary for the DNA replication machinery to properly proceed through highly repetitive portions of the genome. This last hypothesis is in line with the observation that siRNA silencing of either the histone-chaperones RBBP4/7 [42] or of SET8 [46,47] impairs S-phase progression.

Although these observations point towards an upstream role of chromatin structure in determining DNA stability, it is also true that genome integrity influences chromatin structure. Local DNA damage affects the epigenetic status of chromatin both in the vicinity of a lesion through phosphorylation, acetylation and ubiquitination of nearby histones, but also globally [51]. In response to local DNA damage, the zinc finger protein KAP1 is phosporylated by the ATM kinase and released from heterochromatin, thus facilitating the access of DNA repair factors to these more compacted regions of the genome and also potentially altering chromatin structure at other sites [51]. Furthermore, as previously mentioned, DNA damage results in redistribution of chromatin associated factors and histone modifiers like SIRT1, possibly leading to profound changes in the transcriptional regulation of genes [34]. Clearly, the relationship between chromatin structure and DNA damage is not unidirectional, but rather a mutual one.

### A network of aging mechanisms

In our search of molecular mechanisms for biological processes we usually look for linear pathways. What we are learning about the interplay between chromatin structure, epigenetic regulation and DNA repair makes it clear that this is not a one-way street and that these processes are likely connected and linked by feedback mechanisms. The most likely scenario is that chromatin structure, epigenetic status, and DNA repair represent nodes of a network of processes involved in protecting  cells from endogenous and exogenous insults, ultimately leading to increased longevity (Figure 1C). Importantly, these processes do not work in isolation, but instead are linked to pathways dedicated to maintain proteostasis such as the heat shock response or autophagy, and hormonal regulation of cellular growth, with the mTOR and IGF-1 pathways, whose role in the regulation of longevity has already been established in mammals [52-54]. In support of a branched network of cellular functions involved in aging, possible connections between the DNA damage, the inhibition of the IGF-I and mTOR pathways have been suggested [55]. This scenario is supported by observations in cells from the ZMPSTE24-/- mouse, a murine model of HGPS in which lamin A processing is impaired. The progeriod ZEMPSTE24-/- mouse shows dramatic alterations in heterochromatin architecture, accompanied by increased DNA damage, by the activation of the authophagic response and by downregulation of the mTOR pathway [32,56].

Aging is a complex process. It is hardly realistic for it to be explained by a single pathway or even a set of closely related pathways. More likely, many diverse cellular functions will contribute to aging and they will do so in a highly inter-dependent manner. The recent investigation of the role of chromatin structure, epigenetic modifications and DNA damage in aging makes this clear. While we are still struggling to understand the precise relationship of these events in the aging process, we are already discovering links to more distantly related events such as signaling pathways and metabolism.  Rather than attempting to explain aging as the consequence of degeneration of single pathways, a conceptual framework consisting of a network of affected processes not only reconciles different, at times contentious hypotheses regarding aging mechanisms, but will ultimately lead to an integrated view of these processes and to a more accurate understanding of the molecular basis of aging.
